# Bis(2,5-dimethyl­anilinium) tetra­chlorido­zincate(II)

**DOI:** 10.1107/S1600536809009982

**Published:** 2009-03-25

**Authors:** Sofiane Souissi, Wajda Smirani, Mohamed Rzaigui

**Affiliations:** a Laboratoire de Chimie des Matériaux, Faculté des Sciences de Bizerte, 7021 Zarzouna Bizerte, Tunisia

## Abstract

In the title compound, (C_8_H_12_N)_2_[ZnCl_4_], the Zn^2+^ ion adopts a distorted tetra­hedral coordination geometry. In the crystal, the cations and anions are linked by N—H⋯Cl hydrogen bonds, leading to ribbons propagating parallel to the *a* axis.

## Related literature

For related structures, see: Guo *et al.* (2007 ▶); Smirani & Rzaigui (2009 ▶). For background on hybrid materials, see: Tao *et al.* (2003 ▶); Bringley & Rajeswaran (2006 ▶).
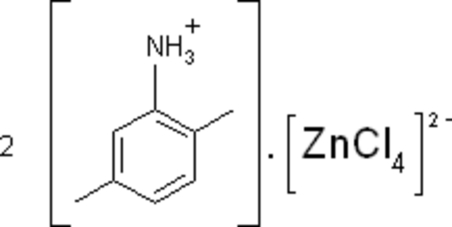


## Experimental

### 

#### Crystal data

(C_8_H_12_N)_2_[ZnCl_4_]
*M* *_r_* = 451.58
Monoclinic, 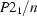

*a* = 7.425 (2) Å
*b* = 12.884 (2) Å
*c* = 22.809 (2) Å
β = 96.16 (2)°
*V* = 2169.5 (7) Å^3^
*Z* = 4
Mo *K*α radiation
μ = 1.62 mm^−1^
*T* = 293 K
0.20 × 0.13 × 0.10 mm

#### Data collection

Enraf–Nonius Turbo CAD-4 diffractometer
Absorption correction: none
6539 measured reflections
3947 independent reflections
2621 reflections with *I* > 2σ(*I*)
*R*_int_ = 0.033
2 standard reflections frequency: 120 min intensity decay: 5%

#### Refinement

*R*[*F* ^2^ > 2σ(*F* ^2^)] = 0.079
*wR*(*F* ^2^) = 0.229
*S* = 1.05
3947 reflections
214 parameters
H-atom parameters not refined
Δρ_max_ = 0.50 e Å^−3^
Δρ_min_ = −0.61 e Å^−3^

### 

Data collection: *CAD-4 EXPRESS* (Enraf–Nonius, 1994 ▶); cell refinement: *CAD-4 EXPRESS*; data reduction: *XCAD4* (Harms & Wocadlo, 1995 ▶); program(s) used to solve structure: *SHELXS97* (Sheldrick, 2008 ▶); program(s) used to refine structure: *SHELXL97* (Sheldrick, 2008 ▶); molecular graphics: *ORTEP-3 for Windows* (Farrugia, 1997 ▶); software used to prepare material for publication: *WinGX* (Farrugia, 1999 ▶).

## Supplementary Material

Crystal structure: contains datablocks I, global. DOI: 10.1107/S1600536809009982/hb2930sup1.cif

Structure factors: contains datablocks I. DOI: 10.1107/S1600536809009982/hb2930Isup2.hkl

Additional supplementary materials:  crystallographic information; 3D view; checkCIF report

## Figures and Tables

**Table 1:** Selected bond lengths (Å)

Zn1—Cl1	2.248 (2)
Zn1—Cl2	2.2502 (16)
Zn1—Cl3	2.274 (2)
Zn1—Cl4	2.2721 (18)

**Table 2:** Hydrogen-bond geometry (Å, °)

*D*—H⋯*A*	*D*—H	H⋯*A*	*D*⋯*A*	*D*—H⋯*A*
N1—H1*A*⋯Cl4^i^	0.89	2.25	3.125 (6)	169
N1—H1*B*⋯Cl3^ii^	0.89	2.54	3.304 (6)	145
N1—H1*C*⋯Cl2^iii^	0.89	2.31	3.172 (7)	162
N2—H2*A*⋯Cl1^i^	0.89	2.34	3.219 (6)	171
N2—H2*B*⋯Cl4^iv^	0.89	2.70	3.505 (7)	151
N2—H2*C*⋯Cl3^ii^	0.89	2.39	3.262 (6)	168

Symmetry codes: (i) 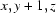; (ii) 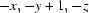; (iii) 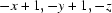; (iv) 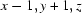.

**Fig. 1.**
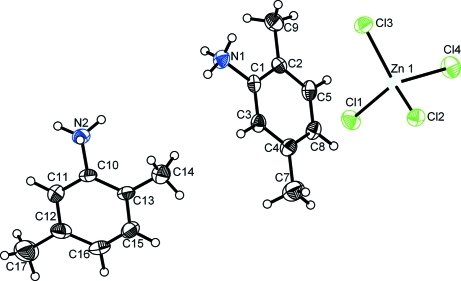
View of the molecular structure of (I): displacement ellipsoids for non-H atoms are drawn at the 30% probability level.

**Fig. 2.**
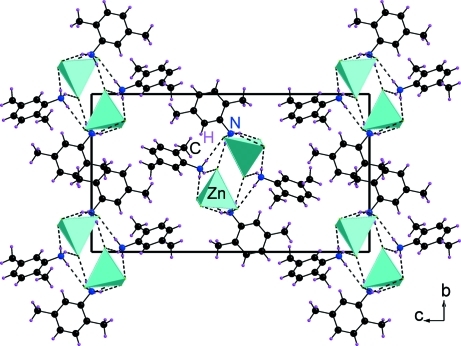
A view of the packing in (I) viewed along the a axis.

## supplementary crystallographic information

### Comment

Inorganic-organic hybrid materials are of great interest in solid state chemistry due to their enormous variety of intriguing structural topologies and their fascinating properties as well as great potential applications in many fields (Tao *et al.*, 2003; Bringley & Rajeswaran, 2006). Here we report the crystal structure of bis(2,5-xylidinium) tetrachlorozincate (I).

As shown in Fig. 1, the asymmetric unit of (I) is built up from two 2,5-xylidinium cations and one tetrachlorozincate (II) anion. The Zn (II) ion is in a tetrahedral coordination environment composed of four Cl anions (Table 1).  The Cl—Zn—Cl bond angles range from 106.13 (8) to 112.46 (8)°. These values indicate that the anionic [ZnCl_4_]^2-^ tetrahedron is slightly distorted (Guo *et al.*, 2007). The examination of the organic cation shows that the values of the N—C, C—C distances and N—C—C, C—C—C angles range from 1.343 (12) to 1.512 (11) Å and 115.90 (7) to 123.0 (6)°, respectively. These values show no significant difference from those obtained in other crystals involving the same organic groups (Smirani and Rzaigui, 2009).

A projection of the structure along the direction a shows that the [ZnCl_4_]^2-^ anions are connected *via* N—H···Cl hydrogen bonds originating from NH_3_^+^ groups, so as to built inorganic ribbons at *x* = 0 and *x* = 1/2 (Fig. 2, Table 2). The 2,5-xylidinium cations are anchored onto the successive ribbons *via* hydrogen bonds and electrostatic and van der Waals interactions, to compensate their negative charges.

### Experimental

An aqueous solution of 2,5-xylidine, HCl and ZnCl_2_ in a 2:2:1 molar ratio was prepared and colourless blocks of (I) grew as the water evaporated over the course of a few days.

### Refinement

All H atoms were positioned geometrically and treated as riding on their parent atoms: N–H = 0.89, C–H = 0.93–0.96 Å and *U*_iso_(H) = 1.2U_eq_(C) or 1.5U_eq_(methyl-C,N).

### Crystal data

**Table:**

(C_8_H_12_N)_2_[ZnCl_4_]	*F*(000) = 928
*M**_r_* = 451.58	*D*_x_ = 1.383 Mg m^−^^3^
Monoclinic, *P*2_1_/*n*	Mo *K*α radiation, λ = 0.71073 Å
Hall symbol: -P 2yn	Cell parameters from 25 reflections
*a* = 7.425 (2) Å	θ = 9.9–11.0°
*b* = 12.884 (2) Å	µ = 1.62 mm^−^^1^
*c* = 22.809 (2) Å	*T* = 293 K
β = 96.16 (2)°	Block, colourless
*V* = 2169.5 (7) Å^3^	0.20 × 0.13 × 0.10 mm
*Z* = 4	

### Data collection

**Table:**

Enraf–Nonius Turbo CAD-4 diffractometer	θ_max_ = 28.0°, θ_min_ = 2.4°
Radiation source: Enraf Nonius FR590	*h* = −9→9
Nonprofiled ω scans	*k* = 0→17
6539 measured reflections	*l* = −10→17
3947 independent reflections	2 standard reflections every 120 min
2621 reflections with *I* > 2σ(*I*)	intensity decay: 5%
*R*_int_ = 0.033	

### Refinement

**Table:**

Refinement on *F*^2^	Primary atom site location: structure-invariant direct methods
Least-squares matrix: full	Secondary atom site location: difference Fourier map
*R*[*F*^2^ > 2σ(*F*^2^)] = 0.079	Hydrogen site location: inferred from neighbouring sites
*wR*(*F*^2^) = 0.229	H-atom parameters not refined
*S* = 1.05	*w* = 1/[σ^2^(*F*_o_^2^) + (0.1531*P*)^2^] where *P* = (*F*_o_^2^ + 2*F*_c_^2^)/3
3947 reflections	(Δ/σ)_max_ = 0.012
214 parameters	Δρ_max_ = 0.50 e Å^−^^3^
0 restraints	Δρ_min_ = −0.61 e Å^−^^3^

### Special details

**Table:**

Geometry. All e.s.d.'s (except the e.s.d. in the dihedral angle between two l.s. planes) are estimated using the full covariance matrix. The cell e.s.d.'s are taken into account individually in the estimation of e.s.d.'s in distances, angles and torsion angles; correlations between e.s.d.'s in cell parameters are only used when they are defined by crystal symmetry. An approximate (isotropic) treatment of cell e.s.d.'s is used for estimating e.s.d.'s involving l.s. planes.
Refinement. Refinement of *F*^2^ against ALL reflections. The weighted *R*-factor *wR* and goodness of fit *S* are based on *F*^2^, conventional *R*-factors *R* are based on *F*, with *F* set to zero for negative *F*^2^. The threshold expression of *F*^2^ > σ(*F*^2^) is used only for calculating *R*-factors(gt) *etc*. and is not relevant to the choice of reflections for refinement. *R*-factors based on *F*^2^ are statistically about twice as large as those based on *F*, and *R*- factors based on ALL data will be even larger.

### Fractional atomic coordinates and isotropic or equivalent isotropic displacement parameters (Å^2^)

**Table:**

	*x*	*y*	*z*	*U*_iso_*/*U*_eq_	
Cl1	0.0360 (2)	0.16857 (16)	0.12060 (10)	0.0688 (6)	
Cl2	0.52806 (19)	0.20922 (13)	0.10654 (9)	0.0537 (5)	
Cl3	0.1904 (2)	0.22519 (13)	−0.02438 (9)	0.0583 (6)	
Cl4	0.3103 (3)	−0.02537 (13)	0.04807 (11)	0.0808 (7)	
N1	0.2378 (7)	0.7499 (4)	−0.0004 (3)	0.0562 (16)	
H1A	0.2629	0.8096	0.0183	0.084*	
H1B	0.1189	0.7451	−0.0105	0.084*	
H1C	0.2950	0.7474	−0.0328	0.084*	
C1	0.2981 (7)	0.6631 (4)	0.0386 (3)	0.0414 (17)	
C3	0.3769 (8)	0.6844 (4)	0.0940 (3)	0.0490 (19)	
H3	0.3922	0.7531	0.1061	0.059*	
C4	0.4345 (8)	0.6053 (5)	0.1327 (3)	0.0527 (18)	
C2	0.2704 (7)	0.5621 (4)	0.0171 (3)	0.0407 (16)	
C5	0.3295 (8)	0.4834 (4)	0.0569 (3)	0.0513 (19)	
H5	0.3153	0.4146	0.0451	0.062*	
C9	0.1839 (10)	0.5398 (6)	−0.0438 (3)	0.062 (2)	
H9A	0.0653	0.5703	−0.0490	0.093*	
H9B	0.1742	0.4661	−0.0495	0.093*	
H9C	0.2567	0.5686	−0.0721	0.093*	
C8	0.4080 (9)	0.5049 (5)	0.1130 (4)	0.0530 (19)	
H8	0.4441	0.4502	0.1382	0.064*	
C7	0.5214 (14)	0.6282 (7)	0.1943 (4)	0.086 (3)	
H7A	0.6431	0.6522	0.1925	0.129*	
H7B	0.5235	0.5661	0.2177	0.129*	
H7C	0.4529	0.6808	0.2119	0.129*	
N2	−0.2368 (8)	0.9737 (4)	0.1100 (3)	0.0565 (17)	
H2A	−0.1525	1.0231	0.1114	0.085*	
H2B	−0.3429	0.9999	0.0952	0.085*	
H2C	−0.2061	0.9220	0.0872	0.085*	
C10	−0.2512 (9)	0.9346 (4)	0.1696 (3)	0.0456 (17)	
C13	−0.1163 (9)	0.8706 (5)	0.1964 (3)	0.0483 (19)	
C11	−0.3993 (9)	0.9637 (6)	0.1974 (4)	0.062 (2)	
H11	−0.4863	1.0074	0.1782	0.075*	
C14	0.0465 (11)	0.8399 (7)	0.1675 (4)	0.082 (3)	
H14A	0.0186	0.7799	0.1433	0.123*	
H14B	0.1443	0.8240	0.1971	0.123*	
H14C	0.0813	0.8961	0.1434	0.123*	
C12	−0.4195 (10)	0.9281 (7)	0.2541 (4)	0.065 (2)	
C16	−0.2860 (11)	0.8619 (6)	0.2797 (4)	0.062 (2)	
H16	−0.2972	0.8346	0.3169	0.075*	
C15	−0.1412 (11)	0.8360 (6)	0.2523 (4)	0.062 (2)	
H15	−0.0538	0.7929	0.2718	0.075*	
C17	−0.5803 (14)	0.9593 (10)	0.2849 (5)	0.120 (4)	
H17A	−0.6893	0.9349	0.2627	0.180*	
H17B	−0.5844	1.0335	0.2879	0.180*	
H17C	−0.5701	0.9293	0.3236	0.180*	
Zn1	0.26302 (8)	0.14604 (5)	0.06412 (4)	0.0418 (3)	

### Atomic displacement parameters (Å^2^)

**Table:**

	*U*^11^	*U*^22^	*U*^33^	*U*^12^	*U*^13^	*U*^23^
Cl1	0.0506 (9)	0.0874 (13)	0.0709 (18)	0.0007 (8)	0.0174 (9)	−0.0083 (11)
Cl2	0.0423 (7)	0.0579 (9)	0.0584 (15)	−0.0086 (6)	−0.0064 (7)	−0.0055 (8)
Cl3	0.0709 (10)	0.0511 (9)	0.0506 (16)	−0.0023 (7)	−0.0040 (9)	0.0047 (8)
Cl4	0.1136 (16)	0.0354 (8)	0.0909 (19)	−0.0001 (9)	−0.0004 (13)	−0.0092 (9)
N1	0.067 (3)	0.039 (3)	0.063 (5)	0.003 (2)	0.004 (3)	0.008 (3)
C1	0.036 (3)	0.033 (3)	0.056 (6)	0.003 (2)	0.012 (3)	0.003 (3)
C3	0.047 (3)	0.036 (3)	0.063 (6)	0.000 (2)	0.001 (3)	−0.004 (3)
C4	0.048 (3)	0.052 (3)	0.057 (6)	−0.001 (3)	−0.001 (3)	0.004 (3)
C2	0.037 (3)	0.041 (3)	0.046 (5)	−0.004 (2)	0.012 (3)	−0.001 (3)
C5	0.047 (3)	0.033 (3)	0.076 (6)	−0.001 (2)	0.011 (3)	0.005 (3)
C9	0.066 (4)	0.056 (4)	0.064 (7)	−0.008 (3)	0.006 (4)	−0.012 (4)
C8	0.053 (3)	0.042 (3)	0.063 (6)	0.005 (3)	0.000 (4)	0.008 (3)
C7	0.101 (7)	0.075 (5)	0.077 (8)	−0.001 (5)	−0.014 (6)	0.001 (5)
N2	0.075 (4)	0.054 (3)	0.041 (5)	0.004 (3)	0.005 (3)	0.008 (3)
C10	0.060 (4)	0.047 (3)	0.029 (5)	−0.006 (3)	0.000 (3)	0.001 (3)
C13	0.057 (4)	0.053 (3)	0.033 (6)	−0.003 (3)	−0.004 (3)	0.001 (3)
C11	0.058 (4)	0.069 (4)	0.058 (7)	0.013 (3)	−0.003 (4)	0.000 (4)
C14	0.068 (5)	0.109 (7)	0.067 (8)	0.026 (5)	0.002 (5)	−0.002 (5)
C12	0.067 (4)	0.087 (5)	0.042 (7)	−0.006 (4)	0.012 (4)	−0.002 (4)
C16	0.075 (5)	0.076 (5)	0.034 (6)	−0.009 (4)	−0.001 (4)	0.009 (4)
C15	0.068 (5)	0.065 (4)	0.051 (7)	0.006 (3)	−0.008 (4)	0.007 (4)
C17	0.085 (6)	0.195 (13)	0.082 (9)	0.028 (7)	0.020 (6)	−0.011 (8)
Zn1	0.0383 (4)	0.0382 (4)	0.0481 (8)	−0.0011 (3)	0.0007 (3)	−0.0031 (3)

### Geometric parameters (Å, °)

**Table:**

Zn1—Cl1	2.248 (2)	C7—H7B	0.9600
Zn1—Cl2	2.2502 (16)	C7—H7C	0.9600
Zn1—Cl3	2.274 (2)	N2—C10	1.463 (9)
Zn1—Cl4	2.2721 (18)	N2—H2A	0.8900
N1—C1	1.468 (8)	N2—H2B	0.8900
N1—H1A	0.8900	N2—H2C	0.8900
N1—H1B	0.8900	C10—C11	1.379 (10)
N1—H1C	0.8900	C10—C13	1.388 (9)
C1—C3	1.364 (10)	C13—C15	1.382 (11)
C1—C2	1.399 (8)	C13—C14	1.491 (11)
C3—C4	1.386 (9)	C11—C12	1.396 (11)
C3—H3	0.9300	C11—H11	0.9300
C4—C8	1.377 (9)	C14—H14A	0.9600
C4—C7	1.512 (11)	C14—H14B	0.9600
C2—C5	1.400 (9)	C14—H14C	0.9600
C2—C9	1.495 (10)	C12—C16	1.388 (11)
C5—C8	1.376 (10)	C12—C17	1.503 (13)
C5—H5	0.9300	C16—C15	1.343 (12)
C9—H9A	0.9600	C16—H16	0.9300
C9—H9B	0.9600	C15—H15	0.9300
C9—H9C	0.9600	C17—H17A	0.9600
C8—H8	0.9300	C17—H17B	0.9600
C7—H7A	0.9600	C17—H17C	0.9600
			
C1—N1—H1A	109.5	C10—N2—H2C	109.5
C1—N1—H1B	109.5	H2A—N2—H2C	109.5
H1A—N1—H1B	109.5	H2B—N2—H2C	109.5
C1—N1—H1C	109.5	C11—C10—C13	122.2 (7)
H1A—N1—H1C	109.5	C11—C10—N2	118.4 (6)
H1B—N1—H1C	109.5	C13—C10—N2	119.5 (6)
C3—C1—C2	123.0 (6)	C15—C13—C10	115.9 (7)
C3—C1—N1	118.8 (5)	C15—C13—C14	121.2 (7)
C2—C1—N1	118.2 (6)	C10—C13—C14	122.9 (7)
C1—C3—C4	121.0 (6)	C10—C11—C12	120.5 (7)
C1—C3—H3	119.5	C10—C11—H11	119.8
C4—C3—H3	119.5	C12—C11—H11	119.8
C8—C4—C3	117.4 (7)	C13—C14—H14A	109.5
C8—C4—C7	121.2 (7)	C13—C14—H14B	109.5
C3—C4—C7	121.3 (7)	H14A—C14—H14B	109.5
C1—C2—C5	115.0 (6)	C13—C14—H14C	109.5
C1—C2—C9	122.5 (6)	H14A—C14—H14C	109.5
C5—C2—C9	122.5 (6)	H14B—C14—H14C	109.5
C8—C5—C2	122.0 (6)	C16—C12—C11	116.7 (7)
C8—C5—H5	119.0	C16—C12—C17	122.3 (9)
C2—C5—H5	119.0	C11—C12—C17	121.0 (8)
C2—C9—H9A	109.5	C15—C16—C12	121.8 (8)
C2—C9—H9B	109.5	C15—C16—H16	119.1
H9A—C9—H9B	109.5	C12—C16—H16	119.1
C2—C9—H9C	109.5	C16—C15—C13	122.9 (7)
H9A—C9—H9C	109.5	C16—C15—H15	118.5
H9B—C9—H9C	109.5	C13—C15—H15	118.5
C5—C8—C4	121.6 (6)	C12—C17—H17A	109.5
C5—C8—H8	119.2	C12—C17—H17B	109.5
C4—C8—H8	119.2	H17A—C17—H17B	109.5
C4—C7—H7A	109.5	C12—C17—H17C	109.5
C4—C7—H7B	109.5	H17A—C17—H17C	109.5
H7A—C7—H7B	109.5	H17B—C17—H17C	109.5
C4—C7—H7C	109.5	Cl1—Zn1—Cl2	112.46 (8)
H7A—C7—H7C	109.5	Cl1—Zn1—Cl4	110.87 (9)
H7B—C7—H7C	109.5	Cl2—Zn1—Cl4	106.13 (8)
C10—N2—H2A	109.5	Cl1—Zn1—Cl3	109.26 (8)
C10—N2—H2B	109.5	Cl2—Zn1—Cl3	109.42 (7)
H2A—N2—H2B	109.5	Cl4—Zn1—Cl3	108.59 (9)
			
C2—C1—C3—C4	−0.1 (9)	C11—C10—C13—C15	1.3 (10)
N1—C1—C3—C4	179.4 (6)	N2—C10—C13—C15	−179.2 (6)
C1—C3—C4—C8	−0.4 (10)	C11—C10—C13—C14	−178.8 (7)
C1—C3—C4—C7	−179.8 (7)	N2—C10—C13—C14	0.7 (10)
C3—C1—C2—C5	0.1 (8)	C13—C10—C11—C12	−0.7 (11)
N1—C1—C2—C5	−179.3 (5)	N2—C10—C11—C12	179.7 (6)
C3—C1—C2—C9	−180.0 (6)	C10—C11—C12—C16	−1.0 (11)
N1—C1—C2—C9	0.6 (9)	C10—C11—C12—C17	180.0 (9)
C1—C2—C5—C8	0.3 (9)	C11—C12—C16—C15	2.2 (12)
C9—C2—C5—C8	−179.6 (6)	C17—C12—C16—C15	−178.7 (9)
C2—C5—C8—C4	−0.8 (10)	C12—C16—C15—C13	−1.8 (12)
C3—C4—C8—C5	0.8 (10)	C10—C13—C15—C16	0.0 (11)
C7—C4—C8—C5	−179.8 (7)	C14—C13—C15—C16	−179.9 (8)

### Hydrogen-bond geometry (Å, °)

**Table:**

*D*—H···*A*	*D*—H	H···*A*	*D*···*A*	*D*—H···*A*
N1—H1A···Cl4^i^	0.89	2.25	3.125 (6)	169
N1—H1B···Cl3^ii^	0.89	2.54	3.304 (6)	145
N1—H1C···Cl2^iii^	0.89	2.31	3.172 (7)	162
N2—H2A···Cl1^i^	0.89	2.34	3.219 (6)	171
N2—H2B···Cl4^iv^	0.89	2.70	3.505 (7)	151
N2—H2C···Cl3^ii^	0.89	2.39	3.262 (6)	168

Symmetry codes: (i) *x*, *y*+1, *z*; (ii) −*x*, −*y*+1, −*z*; (iii) −*x*+1, −*y*+1, −*z*; (iv) *x*−1, *y*+1, *z*.

## References

[bb1] Bringley, J. F. & Rajeswaran, M. (2006). *Acta Cryst.* E**62**, m1304–m1305.

[bb2] Enraf–Nonius (1994). *CAD-4 EXPRESS* Enraf–Nonius, Delft, The Netherlands.

[bb3] Farrugia, L. J. (1997). *J. Appl. Cryst.* **30**, 565.

[bb4] Farrugia, L. J. (1999). *J. Appl. Cryst.* **32**, 837–838.

[bb5] Guo, N., Yi, J., Chen, Y., Liao, S. & Fu, Z. (2007). *Acta Cryst. *E**63, **m2571.

[bb6] Harms, K. & Wocadlo, S. (1995). *XCAD4* University of Marburg, Germany.

[bb7] Sheldrick, G. M. (2008). *Acta Cryst.* A**64**, 112–122. 10.1107/S0108767307043930 18156677

[bb8] Smirani, W. & Rzaigui, M. (2009). *Acta Cryst.* E**65**, o83. 10.1107/S1600536808041287 PMC2967991 21581721

[bb9] Tao, J., Yin, X., Jiang, Y. B., Yang, L. F., Huang, R. B. & Zheng, L. S. (2003). *Eur. J. Inorg. Chem.* pp. 2678–2682.

